# Effect of Cricket Frass Fertilizer on growth and pod production of green beans (*Phaseolus vulgaris* L.)

**DOI:** 10.1371/journal.pone.0303080

**Published:** 2024-05-09

**Authors:** Clarcky Andrianorosoa Ony, Cédrique L. Solofondranohatra, Tanjona Ramiadantsoa, Andrianjaka Ravelomanana, Nivohanintsoa Ramanampamonjy R., Sylvain Hugel, Brian L. Fisher

**Affiliations:** 1 Madagascar Biodiversity Center, Antananarivo, Madagascar; 2 Mention Entomologie Cultures Elevage et Santé, Faculté des Sciences, Université d’Antananarivo, Antananarivo, Madagascar; 3 Mention Biologie Ecologie Végétales, Faculté des Sciences, Université d’Antananarivo, Antananarivo, Madagascar; 4 Institut des Neurosciences Cellulaires et Intégratives, Centre National de la Recherche Scientifique, Université de Strasbourg, Strasbourg, France; 5 Department of Entomology, California Academy of Sciences, San Francisco, California, United States of America; Universita degli Studi della Basilicata, ITALY

## Abstract

Cricket Frass Fertilizer (CFF) was tested for its efficiency and potential as a fertilizer on the growth of green beans (*Phaseolus vulgaris* L.) in central Madagascar from April 2020 to October 2020. We grew green beans experimentally for 93 days with seven different fertilizer treatments: NPK 200 kg/ha (0.47 g of N/plant), GUANOMAD (guano from bat) 300 kg/ha (0.26 g of N/ plant), CFF 100 kg/ha (0.12 g of N/plant), CFF 200 kg/ha (0.24 g of N/plant), CFF 300 kg/ha (0.38 g of N/plant), CFF 400 kg/ha (0.52 g of N/plant), and no fertilizer (0 g of N/plant). Three plant traits were measured: survival proportion, vegetative biomass, and pod biomass. The survival proportion of plants treated with the highest dose of CFF (400 kg/ha, 88.1%), NPK (79.8%), and GUANOMAD (81.2%) were similar, but plants treated with the former yielded significantly higher vegetative (35.5 g/plant) and pod biomass (11 g/plant). These results suggest that fertilizing green beans with CFF at a 400 kg/ha dose is sufficient for plant survival and growth, and improves pod production. In Madagascar where soil quality is poor, dependence on imported chemical fertilizers (NPK) and other organic fertilizer (GUANOMAD) can be reduced. Cricket Frass Fertilizer can be used as an alternative sustainable fertilizer for beans.

## Introduction

One major current global challenge is to ensure food security to sustain a dramatically growing population while reducing the negative impacts of agriculture on the environment [1, 2]. Low soil fertility is a major constraint to improving agricultural production, making fertilizer use critical [3]. However, excessive use of chemical fertilizer to increase yields causes unrecoverable environmental footprints [4, 5]. Amongst several issues, chemical fertilizer use reduces biodiversity [6], pollutes marine and terrestrial ecosystems [4, 7], and leads to soil acidification and compaction [5]. Hence, sustainable agricultural production requires the adoption of alternative agricultural practices such as the use of organic fertilizers [8]. The use of organic soil amendments represents an environmentally friendly and economically viable way to increase soil fertility and improve crop yield [9, 10], as well as recycle organic waste products sustainably [11].

The growing practice of farming insects for food and feed [12] offers a new source of organic fertilizer. Insect manure, or frass, is by definition “a mixture of excrements derived from farmed insects, the feeding substrate, parts of farmed insects, dead eggs and with a content of dead farmed insects of not more than 5% in volume and not more than 3% in weight” (Commission Regulation (EU) 2021/192, [13]). Frass has been shown to be an effective organic fertilizer with the capacity to supply nutrients to plants, and the potential to replace conventional fertilizers [14, 15]. Many studies advocate the use of insect frass as a soil amendment that improves soil quality and aids plant growth [12, 16]. These benefits are largely attributed to the nitrogen-rich properties of insect frass [17–20]. Amending soil with insect frass also stimulates soil microbial activity, which reduces plant sensitivity to pathogens, and produces secondary metabolites to protect plants against pathogens [10, 14, 15], all of which enhance plant growth. Therefore, the use of insect frass as fertilizer represents an opportunity to support sustainable crop production [21].

Unlike the frass of mealworms (*Tenebrio molitor* L.) and black soldier flies (*Hermetia illucens* L.), which have been the object of more numerous studies referring to their fertilizing potential, few studies have explored the use of cricket frass to amend soils for crops [22–24]. Butnan et al. (2022) showed that combining cricket frass with *Eucalyptus* biochar improved the yield of Chinese kale (*Brassica oleracea* L.). An experiment comparing the growth of spider plant (*Cleome gynandra* L.) supplemented with cricket frass, cow manure, and synthetic fertilizer showed that frass increased available nitrogen in the soil, and improved vegetative growth [24]. Ferruzca-Campos et al. (2023) demonstrated that substrate amended with less than 1% (w/w) cricket frass, that contains 4.035% of total nitrogen significantly improved the growth and development of tomato plants.

In the highlands of Madagascar, soils are cropped intensively, which has led to a decrease in fertility and soil organic carbon contents [25, 26]. Appropriate fertilizer management strategies are required to improve crop production in the system, yet organic resources are the primary fertilizers accessible to Malagasy farmers [27]. Cricket frass fertilizer (CFF), a new organic fertilizer from local farming of the cricket species *Gryllus madagascarensis* [28], has become available in the country. No study has yet assessed the potential role of CFF on crop growth in Madagascar. Here, we aim to determine the efficiency of CFF as a fertilizer in Madagascar’s agroclimatological conditions. This marks the first study exploring the effect of CFF on plants in Madagascar. We compared the performance of CFF against the chemical fertilizer NPK and the organic fertilizer GUANOMAD (guano derived from bat feces) on the survival, growth, and pod production of green bean plants. Green beans were chosen because, after tomatoes, they are the second-most cultivated vegetable in the commune where the experiment was carried out, occupying about 21% of the vegetable cropping area [29].

## Materials and methods

### Experimental site characteristics

The experiment was conducted in Ampangabe Commune, Ambohidratrimo District, Analamanga Region (18.859° S, 47.403° E) during the dry season (from April to October 2020). The timing of the planting followed the recommendation of the local horticulture institute (Centre Technique Horticole d’Antananarivo, CTHA). Total average annual rainfall was 1850 mm, with only 15% falling during the dry season [30]. Temperatures ranged from 15°C to 23°C [29]. Soil type was clay (details on soil characteristics are presented in S1 Appendix).

No permit was required to conduct the study as it was done on a private land where only the owner’s approval was needed.

### Fertilizer and crops

This study examined how green bean (*Phaseolus vulgaris* L.), purchased from a local store, responds to fertilizer treatments. We used three fertilizers in the study. The chemical fertilizer NPK 11-22-16 and an organic fertilizer GUANOMAD (N: 4%, P: 13%, K: 2%, GUANOMAD Madagascar company), can both be bought in local stores in Madagascar. Cricket Frass Fertilizer (CFF, Valala Farm Research Lab at MBC, Antananarivo, Madagascar), a mixture of cricket feces and feed residue, is a byproduct of *Gryllus madagascarensis* rearing. The frass was not sifted prior to application. We analyzed the CFF to determine its macro- and micronutrient content (S2 Appendix).

### Treatments and experimental design

In our experiment, we compared the effect of one control (no fertilizer), one dose of NPK (200 kg/ha; following the Centre Technique Horticole d’Antananarivo recommendation for green beans), one dose of GUANOMAD (300 kg/ha; following the GUANOMAD company recommendation for green beans), and four doses of CFF (CFF 100: 100 kg/ha, CFF 200: 200 kg/ha, CFF 300: 300 kg/ha, and CFF 400: 400 kg/ha). The total nitrogen, phosphorus and potassium equivalent per plant for each treatment are given in S3 Appendix. We used four Fisher blocks of 13.5 m x 4.0 m [31]. Each block had twelve plots (3.0 m x 1.0 m) corresponding to three replicates of control, three replicates of NPK, three replicates of GUANOMAD, and three replicates of one of the CFF doses.

Three weeks before sowing, the soil was plowed (40 cm depth) and crumbled. Fourteen green bean seeds were sown in each plot, and fertilizers were applied at planting by adding the appropriate dose in each 15 cm-deep hole. Plants were watered daily. At 93 days after sowing, two measures of plant survival were considered: survival proportions of all individuals (with or without pod), and survival proportions of individuals that produced pods, related to the number of individuals planted. The latter was considered to quantify the treatment effects on the survival of green bean plants that could produce pods. Vegetative biomass (leaves and stems) and pod biomass were harvested and their fresh weight was measured. Plants with pods weighing ≤ 2 g and vegetative biomass ≤ 3 g were excluded from the analysis as these were all dry at 93 days.

### Data analysis

We tested data for normality using the Shapiro-Wilk test. We used the Test of Equal or Given Proportions to determine the differences in plant survival rates among all the treatments. Then, we conducted a pairwise proportion test to compare the difference among treatments. Data on plant biomass were analyzed using the Kruskal-Wallis test. Then, Dunn tests were used for pairwise comparisons. All data analyses were performed in the R environment [32] and the level of significance was set at *p* < 0.05. All data are in S4 Appendix.

## Results

**Fig 1:**
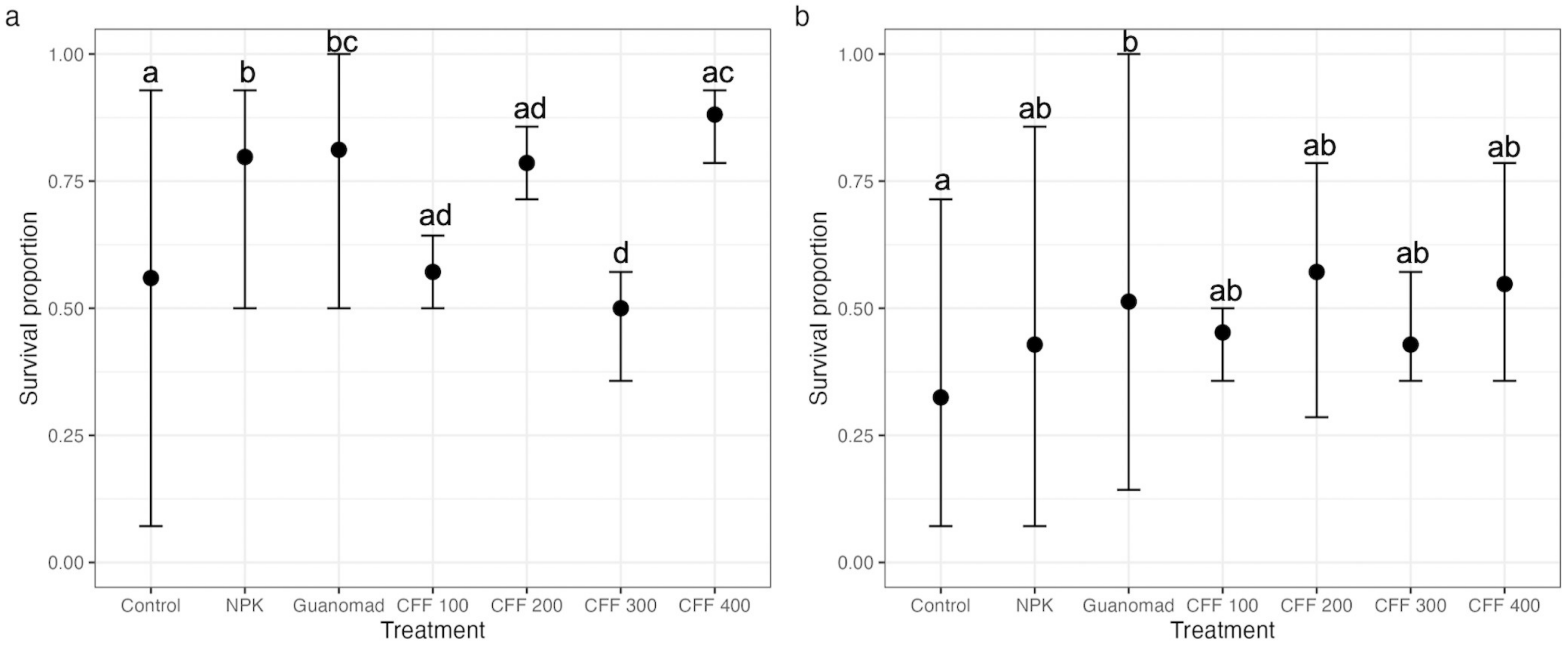
Proportion of surviving green bean plants: (a) all plants (b) plants with pod, compared among treatments. Values are averaged (n = 168 for control, NPK and GUANOMAD; n = 56 for each dose of CFF) with error bars representing maximum and minimum values. Letters indicate significance of difference of survival proportion means between treatment; same letters indicate no significant difference and different letters indicate significant differences at p = 0.05.

Green bean survival proportions of all plants (with or without pod) and plants that produced pods differed significantly among treatments (Proportion test, *p* < 0.001, and *p* = 0.04 respectively, Fig 1). On average, plants treated with NPK (0.47 g of N/ plant) and GUANOMAD (0.26 g of N/ plant) had significantly higher survival proportions (79.8% and 81.2%, respectively) compared with plants treated with CFF 100 (0.12 g of N/ plant), CFF 200 (0.24 g of N/ plant), CFF 300 (0.38 g of N/ plant), or controls (0 g of N/ plant). Among all plants, the highest survival proportion was associated with the CFF 400 (0.52 g of N/ plant) group (88.1%), which was similar to that of the GUANOMAD group and significantly higher than obtained with NPK (Proportion test, *p* = 0.03). There was no significant difference between the survival proportion of plants having produced pods for all treatments except for the control group, which was significantly lower than obtained with GUANOMAD (Proporion test, *p* = 0.001).

**Fig 2:**
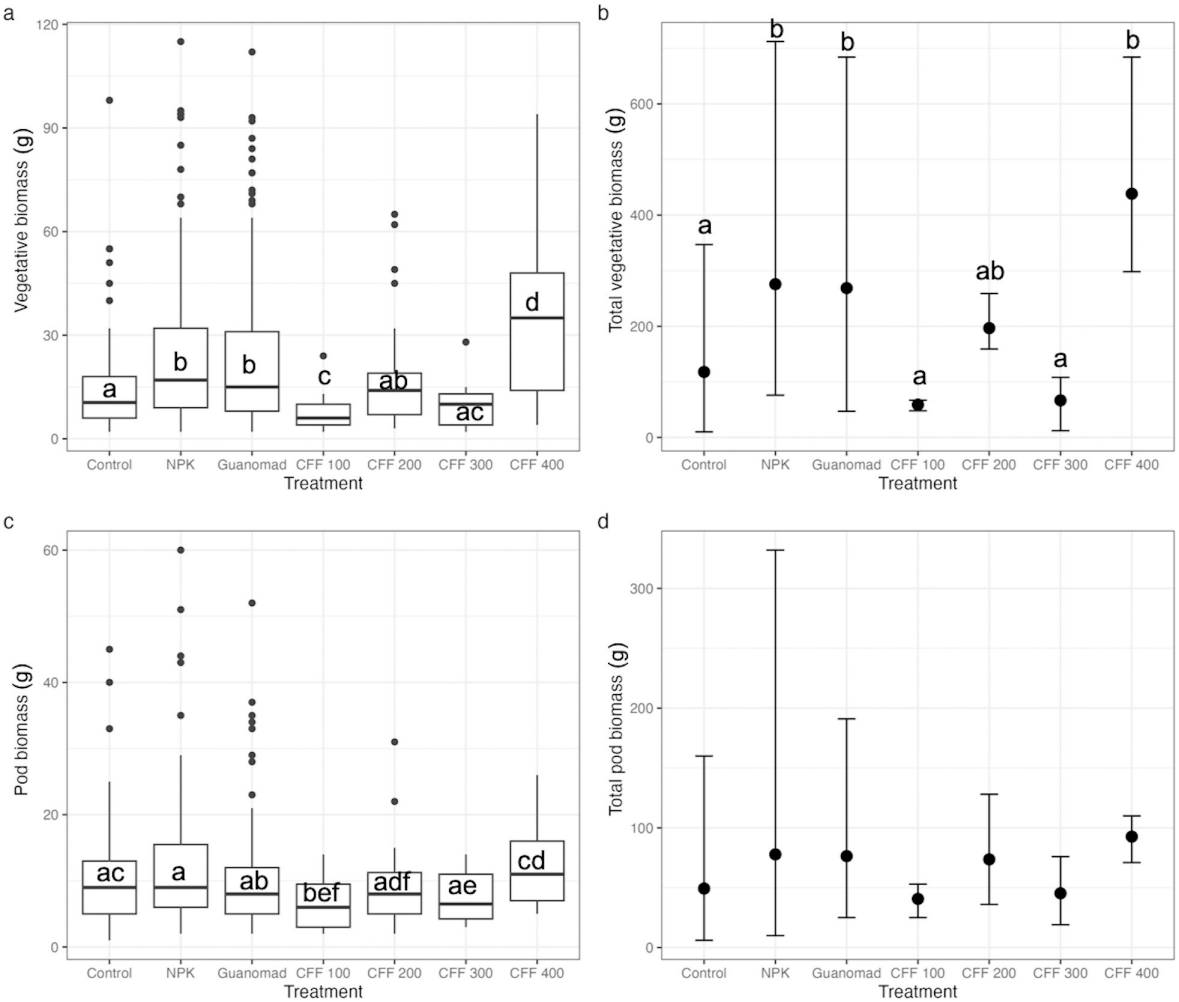
Vegetative biomass of green bean plants (a) per individuals, (b) total per plot; pod biomass (c) per individuals, (d) total per plot, compared among treatments. Values for total vegetative and pod biomass are averaged (n = 168 for control, NPK and GUANOMAD; n = 56 for each dose of CFF) with error bars representing maximum and minimum values. Letters indicate significance of difference of trait means between treatments, with same letters indicating no significant difference and different letters indicating significant differences at p = 0.05.

Plants fertilized with NPK, GUANOMAD, and CFF 400 produced significantly higher vegetative biomass (per plant and total, Fig 2a and 2b) than control plants (Kruskal-Wallis test, all *p* < 0.01 for vegetative biomass per plant; *p* = 0.006, *p* = 0.009, and *p* = 0.003, respectively for total vegetative biomass). The highest average vegetative biomass weight per plant (Fig 2a) was obtained with CFF 400 (35.5 g), which was significantly higher than all the other treatments (Kruskal-Wallis test, *p* < 0.001 for all treatments). The highest total vegetative biomass (Fig 2b) was also obtained with CFF 400 (438 g, with a minimum weight of 298 g and a maximum weight of 684 g), which was similar to the NPK and GUANOMAD groups (Kruskal-Wallis test, *p* = 0.13, and *p* = 0.12, respectively). There was no significant difference between the pod biomass per plant obtained from control, NPK, GUANOMAD, CFF 200 and CFF 300 (Fig 2c). Plants fertilized with CFF 400 produced the highest pod biomass per plant (median = 11 g), which was significantly higher than all the other treatments but the control. Treatments did not have any significant effect on the total pod biomass of green bean plants (Kruskal-Wallis test, *p* = 0.41) but plants fertilized with CFF 400 produced the highest mean value (92.7 g) and the highest minimum value (71 g).

**Fig 3:**
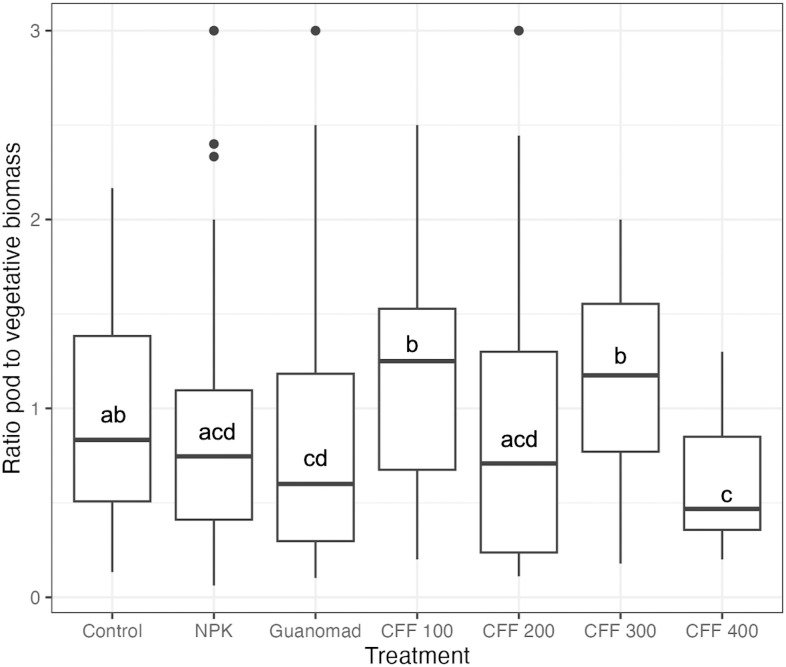
Ratio of pod to vegetative biomass of green beans plants compared among treatments. Letters indicate significance of difference of the ratio means between treatments, with same letters indicating no significant difference and different letters indicating significant differences at p = 0.05.

Treatments significantly affected green bean pod to vegetative biomass ratio (Kruskal-Wallis test, *p* = 0.004). The two highest ratios were obtained from CFF 100 and CFF 300 (Fig 3, median = 1.25 and 1.18, respectively) and were significantly higher than the ratio obtained from NPK and GUANOMAD. The lowest average ratio was associated with CFF 400 (0.67).

## Discussion

We conducted an experiment to quantify the effects of CFF, a new organic fertilizer produced by cricket rearing, on green bean crop survival, growth, and yield. We tested four doses of CFF and compared them with the recommended dose of NPK and GUANOMAD for green bean crops. Demonstrating the potential of CFF to improve green bean production will encourage the integration of this fertilizer into farming practices.

Plant nutrient acquisition is influenced by morphological and physiological characteristics that adjust in response to environmental factors such as climate, soil, and fertilizer application [e.g. 33, 34]. Among the four doses of CFF tested, the highest dose (400 kg/ha) had the most comparable effect to NPK and GUANOMAD on green bean plants. At a dose of 400 kg/ha of CFF, each green bean plant received a total nitrogen of 0.52 g, which is similar to NPK’s and the double of GUANOMAD’s (S3 Appendix). Vegetative and pod biomass production per plant increased with the addition of CFF at 400 kg/ha, which supported previous findings that using insect frass fertilizer improved nutrient levels in soil [e.g. 19, 350, 36]. Organic nitrogen is relatively abundant in most frass ranging between 1.6–7% [37]. This element is essential for plant growth and development, specifically for biomass production [5, 38]. Our results are in line with previous findings demonstrating that the contribution of nitrogen from frass supplied to the soil boosts the growth of plants such as dragon fruit cacti (*Selenicereus undatus* D.R. Hunt) and chards (*Beta vulgaris* L.) [13, 39]. Although the amount of nitrogen provided by GUANOMAD was smaller than that provided by CFF (400 kg/ha) and NPK, it still promoted green beans growth, possibly because it contains microbacterial flora that facilitates nutrient absorption [40]. The amount of nitrogen provided by the other doses of CFF (100 kg/ha, 200 kg/ha, and 300 kg/ha) might not have been sufficient for our plants to grow as much vegetative biomass as those treated with NPK and GUANOMAD (Fig 2a and 2b) but just enough to allocate more biomass to vegetative tissues than pods (Fig 3). It is important to note that green beans, like other legumes species has symbiotic association with bacteria that can fix atmospheric nitrogen [41], and that frass also contains nitrifying bacteria that make nitrogen within the soil accessible to plants [37]. These might have improved green beans nitrogen access and contributed to its vegetative biomass production for plants treated with CFF (Fig 2a and 2b). Frass phytotoxicity is often associated with the addition of high frass concentration [37], which we did not observe at the highest dose of CFF. This suggests that at a dose of 400 kg/ha, CFF does not cause deleterious impact on green bean plant growth and biomass production.

A significantly higher proportion of green bean plants treated with CFF at 400 kg/ha survived than plants grown with NPK, but this figure was similar to that of GUANOMAD (Fig 1a). Organic fertilizers keep plants healthy and suppress some diseases, while chemical fertilizers can increase the sensitivity of plants to diseases and pests [42, 43]. Additionally, insect frass contains chitin, which improves soil quality and plant health by stimulating plant defenses against pathogens [20, 44, 45]. Plants treated with CFF 400 had a higher chance of survival, as insect frass might have increased the abundance of soil microorganisms and might have stimulated their biocontrol activity, which made the crops resistant to pests and diseases [46, 47].

The positive effects of fertilizers on plant growth are attributed primarily to increased nutrient levels in the soil. These effects are generally well understood for fertilizers such as NPK and compost [48–52]. Here, we showed that cricket frass is more effective than NPK and GUANOMAD in promoting the vegetative and pod biomass per plant of green bean plants at an application rate of 400 kg/ha (Fig 2a and 2c), indicating that nutrients for growth in frass are effective in crops. Frass is characterized with high levels of organic carbon [18, 53, 54]. It has been shown that frass decomposes and mineralizes rapidly after its incorporation into the soil [15, 55–57], mainly because of its high carbon content [53]. This might have made nutrients readily available to the green beans during their early growth stages. Phosphorus is one of the most essential nutrients for fruit development [e.g., 5, 58], and is required at an adequate level in the early stages of plant growth [59]. Potassium is another important nutrient that influences plant growth and development [60]. Studies have shown that it protects plants against biotic and abiotic stresses and contributes to their survival [e.g. 61, 62]. Though the amount of phosphorus and potassium supplied by CFF was relatively small compared to NPK (S3 Appendix), it might have been sufficient for the green bean plants to optimize their fruit production and stress tolerance at a dose of 400 kg/ha. Similar results have been found by other authors who demonstrated that applying even relatively low rates of organic amendments increased crop yields by significantly improving soil conditions [35, 63, 64].

This study presents the first example of how cricket frass affects plants in Madagascar. Compared to the frass of other insects, CFF is still poorly studied, and most of our conclusions refer to experiments using the frass of other insects on crops. However, different insect species rely on different feeds and substrates to grow. These factors in turn can significantly affect frass quality [18] and may thus affect plant growth in different ways. This work highlights the need to test the effects of cricket frass on the growth of other crops to determine how well cricket frass supplies nutrients to plants, and what strategies plants use to acquire these nutrients.

## Conclusions

Optimizing crop development is an integral part of effective farming management practices. These practices should be tailored to the nutrient requirements of target crop plants. Here, we demonstrated that cricket frass fertilizer from cricket rearing has potential for use as an organic fertilizer to promote green bean plant growth and survival. If supplied at an adequate rate, cricket frass can be a sustainable fertilizer capable of improving crop yields. Its development and promotion holds promise to enhance the livelihoods of smallhold farmers in places like Madagascar where food security is precarious.

## Supporting information

S1 Appendix Site’s soil characteristics. (DOCX)

S2 Appendix CFF nutrient content. (C: Carbon; Available P: available phosphorous; Soluble P: soluble phosphorus; N tot: total nitrogen; Ca: Calcium; Mg: Magnesium; S: sulfur).
(DOCX)

S3 Appendix Total nitrogen (N), phosphorus (P), and potassium (K) equivalent per plant for each fertilizer treatment. (DOCX)

S4 Appendix Data underlying the results presented in the study. (CSV)
