# Supplementary material for: Effect of Cricket Frass Fertilizer on growth and pod production of green beans (Phaseolus vulgaris L.)
Source: PLoS One. 2024 May 9;19(5):e0303080. doi: 10.1371/journal.pone.0303080 (PMC11081369; doi:10.1371/journal.pone.0303080)
Supplement: S1 Appendix — (DOCX) [file pone.0303080.s001.docx]

**Site’s soil characteristics**

| **pH (H_2_O)** | 4.72 |
| --- | --- |
| **Clay (0-2 µ) %** | 40.9 |
| **Fin silt (2-20 µ) %** | 18.21 |
| **Coarse silt (20-50 µ) %** | 5.63 |
| **Fine sand (50-200 µ) %** | 7.47 |
| **Coarse sand (0,2-2 mm) %** | 27.795 |
| **Total organic carbon (g/kg)** | 27.44 |
| **Organic matter (%)** | 4.7 |
