# Supplementary material for: Effect of Cricket Frass Fertilizer on growth and pod production of green beans (Phaseolus vulgaris L.)
Source: PLoS One. 2024 May 9;19(5):e0303080. doi: 10.1371/journal.pone.0303080 (PMC11081369; doi:10.1371/journal.pone.0303080)
Supplement: S2 Appendix — (C: Carbon; Available P: available phosphorous; Soluble P: soluble phosphorus; N tot: total nitrogen; Ca: Calcium; Mg: Magnesium; S: sulfur). (DOCX) [file pone.0303080.s002.docx]

**CFF nutrient content** (C: Carbon; Available P: available phosphorous; Soluble P: soluble phosphorus; N tot: total nitrogen; Ca: Calcium; Mg: Magnesium; S: sulfur).

| **Macronutrients** | **Test value** | **Micronutrients** | **Test value** |
| --- | --- | --- | --- |
| **C (%)** | 33.7 | **Zn(ppm)** | 118.4 |
| **Available P (%)** | 0.29 | **Fe (%)** | 0.2 |
| **Soluble P (%)** | 0.052 | **Mn (ppm)** | 170 |
| **N tot (%)** | 5.62 | **Na (%)** | 0.26 |
| **Ca (%)** | 1.25 | **K (%)** | 1.42 |
| **Mg (%)** | 0.36 |  |  |
| **S (ppm)** | 518 |  |  |
