# Supplementary material for: Effect of Cricket Frass Fertilizer on growth and pod production of green beans (Phaseolus vulgaris L.)
Source: PLoS One. 2024 May 9;19(5):e0303080. doi: 10.1371/journal.pone.0303080 (PMC11081369; doi:10.1371/journal.pone.0303080)
Supplement: S3 Appendix — (DOCX) [file pone.0303080.s003.docx]

**Total nitrogen (N), phosphorus (P), and potassium (K) equivalent per plant for each fertilizer treatment.**

| **Treatment** | **N (g)** | **P (g)** | **K (g)** |
| --- | --- | --- | --- |
| **Control** | 0 | 0 | 0 |
| **NPK** | 0.47 | 0.94 | 0.68 |
| **GUANOMAD** | 0.26 | 0.84 | 0.13 |
| **CFF 100** | 0.12 | 0.02 | 0.03 |
| **CFF 200** | 0.24 | 0.04 | 0.06 |
| **CFF 300** | 0.38 | 0.06 | 0.09 |
| **CFF 400** | 0.52 | 0.08 | 0.12 |
